# Galactolipid biosynthesis in flowers

**DOI:** 10.1186/1999-3110-54-29

**Published:** 2013-08-30

**Authors:** Yuki Nakamura

**Affiliations:** 1 Institute of Plant and Microbial Biology, Academia Sinica, Taipei, Taiwan; 2 Japan Science and Technology Agency, PRESTO, Saitama, Japan

**Keywords:** *Arabidopsis thaliana*, Digalactosyldiacylglycerol (DGDG), Flower, Galactolipid, Monogalactosyldiacylglycerol (MGDG), *Petunia hybrida*

## Abstract

Phospholipids represent the highly conserved structural basis of biological membranes from bacteria to humans. However, plants and other photoautotrophic organisms are unique in using non-phosphorus galactolipids as primary components of their photosynthetic membranes. In light of the biomass of green tissues as compared with that of the overall plant body and the highly stacked thylakoid membrane structures in chloroplasts, galactolipids are the most abundant membrane lipids on the earth. Historically, the roles of galactolipids have been studied mainly in relation to photosynthesis, and recent advances in molecular biology with Arabidopsis and other model organisms have revealed an essential role of galactolipids in photosynthesis. However, these galactolipids are also abundant in non-photosynthetic organs, especially flowers, which suggests their distinct role apart from photosynthesis. The aim of this mini-review is to describe distinct biochemical properties of flower galactolipids and possible new roles, with a summary of the current understanding of galactolipid biosynthesis in Arabidopsis.

**Electronic supplementary material:**

The online version of this article (doi:10.1186/1999-3110-54-29) contains supplementary material, which is available to authorized users.

## Review

### Introduction

**Figure 1:**
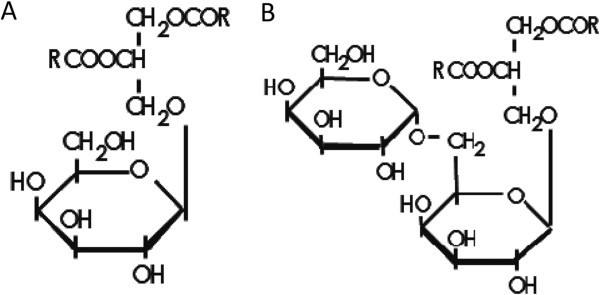
**Chemical structures of (A) MGDG and (B) DGDG.**

Galactolipids are glycoglycerolipids with galactose(s) at the *sn*-3 position of *sn*-1,2-diacylglycerol (DAG; Figure 1; Nakamura et al., 2010). Plant photosynthetic membranes are unique in using galactolipids as a major constituent of biological membranes instead of phospholipids, which most other organisms use. Galactolipids are widely found in photosynthetic organisms such as higher plants, mosses and eukaryotic and prokaryotic algae but are rare in animal and other non-photosynthetic organisms.

The most abundant galactolipid is monogalactosyldiacylglycerol (MGDG), which has one galactose moiety at the *sn*-3 position of the glycerol backbone and constitutes more than 50% of the total chloroplastic membrane lipid (Douce and Joyard, 1980); (Härtel et al., 2000). The second major galactolipid is digalactosyldiacylglycerol (DGDG), which has an additional galactose besides that at the *sn*-3 position of MGDG. MGDG is exclusively localized at plastids, but DGDG can also be found in extraplastidic membranes under conditions such as phosphate deficiency or pollen tube growth (Härtel et al., 2000); (Nakamura et al., 2009); (Botté et al., 2011). The galactolipid biosynthetic pathways localize to the envelope of chloroplasts. The initial substrate DAG is galactosylated by MGDG synthase to produce MGDG. MGDG can be further galactosylated by DGDG synthases to give DGDG. In addition, galactosyltransferase activity can produce oligo GDG (e.g., trigalactosyldiacylglycerol) as well as MGDG and DGDG (van Besouw and Wintermans, 1978); (Moellering et al., 2010).

Initial efforts to understand the biochemical properties of galactolipid biosynthesis involved study of MGDG synthase activity in a range of materials including spinach and cucumber seedlings (Teucher and Heinz, 1991); (Ohta et al., 1995); (Shimojima et al., 1997). Successful purification of native MGDG synthase from cucumber seedlings allowed for molecular biology study in the model system Arabidopsis (Ohta et al., 1995); (Shimojima et al., 1997); (Awai et al., 2001); (Kobayashi et al., 2007); (Kobayashi et al., 2009a). As our understanding of galactolipids in photosynthesis advanced, the function of galactolipids not associated with photosynthesis became an emerging topic. In particular, the finding of significant levels of galactolipids in non-green plastids and active biosynthetic activity during flower development suggested a yet-unknown role of galactolipids (Kleinig and Liedvogel, 1978); (Camara et al., 1983); (Alban et al., 1988); (Nakamura et al., 2003).

After a brief introduction of the current understanding of galactolipid biosynthesis in Arabidopsis, this review summarizes our recent understanding of galactolipids in floral organs. For more extensive information on galactolipid biosynthesis in Arabidopsis, a few detailed reviews have been recently published (Kobayashi et al., 2009b); (Nakamura et al., 2010); (Shimojima and Ohta, 2011).

## Galactolipid biosynthesis in Arabidopsis leaves

**Figure 2:**
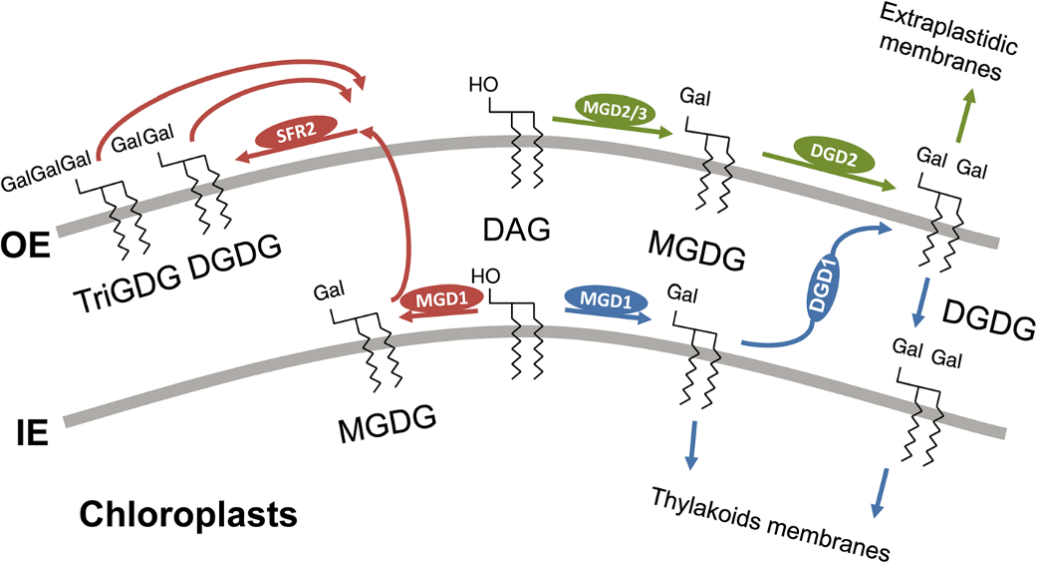
**Three systems of galactolipid biosynthesis in Arabidopsis.** OE, chloroplast outer envelope; IE, chloroplast inner envelope.

Arabidopsis has 3 known galactolipid biosynthesis systems (Figure 2); (Benning and Ohta, 2005). The first system is mediated by MGD1 and DGD1: it is localized at least partially in the inner envelope membrane of chloroplasts and is in charge of the bulk synthesis of the galactolipids MGDG and DGDG. Defects in this pathway severely reduce photosynthetic ability (Dörmann et al., 1995,1999); (Awai et al., 2001); (Froehlich et al., 2001); (Kobayashi et al., 2007). The second system involves MGD2 or MGD3 and DGD2: it is localized at the outer envelope and is conditional (i.e., active with phosphate starvation or in some non-photosynthetic organs such as flowers) (Awai et al., 2001); (Kelly and Dörmann, 2002); (Kelly et al., 2003); (Kobayashi et al., [Bibr CR24][Bibr CR25][Bibr CR27]). The third pathway is by a processive galactosylation that produces MGDG and DGDG and also oligoGDG such as triGDG. Although this activity has been long known biochemically, the gene encoding this enzyme was only recently revealed (Moellering et al., 2010). This activity is also localized at the outer envelope and does not contribute significantly to levels of galactolipids (Dorne et al., 1982); (Kelly et al., 2003); (Moellering et al., 2010).

### MGDG synthases

The most abundant glycerolipid in plants is MGDG, which is synthesized by a galactosyltransferase, UDP-galactose: *sn*-1,2-diacylglycerol 3-β-D-galactosyltransferase (EC 2.4.1.46), or MGDG synthase, with 1,2-*sn*-diacylglycerol and UDP-Gal as substrates (Nakamura et al., 2010). The model plant Arabidopsis has 3 isozymes of MGDG synthases: MGD1, MGD2 and MGD3 (Table 1); (Awai et al., 2001). MGD1 has a chloroplast transit peptide and localizes to the inner envelope of chloroplasts. It is expressed ubiquitously and regulated by light and cytokinin, but MGD1 knockout results in critical defects in photosynthetic growth (Jarvis et al., 2000); (Kobayashi et al., 2007). Thus, MGD1 is called type A MGDG synthase because of its inner envelope localization and fatal effect on photosynthetic growth. MGD2 and MGD3 are type B MGDG synthases because of their contrasting features (Kobayashi et al., 2009a): they have no transit peptides and hence localize at the outer envelope of chloroplasts (Awai et al., 2001); they are mainly expressed in non-photosynthetic organs such as flowers (MGD2) or roots (MGD3) (Awai et al., 2001); (Kobayashi et al., 2004). The expression of type B MGDG synthases is strongly induced by phosphate starvation (Awai et al., 2001); (Kobayashi et al., 2004). This induction also involves auxin signaling, which suggests an auxin–cytokinin crosstalk in regulating the 2 types of MGDG synthases (Kobayashi et al., 2006). Double knockout of MGD2 and MGD3 affects DGDG levels in roots under phosphate starvation, but reduced MGDG level has not been observed in any conditions tested so far (Kobayashi et al., 2009b). MGD1 may be the only isoform that affects MGDG levels in Arabidopsis.

**Table 1:** **Arabidopsis genes encoding galactolipid biosynthetic enzymes**

Enzyme	AGI code	Substrates	Subcellular localization	Mutant phenotype
MGD1	At4g31780	DAG and UDP-Gal	Plastid inner envelope	Greening defect, trace level of MGDG
MGD2	At5g20410	DAG and UDP-Gal	Plastid outer envelope	Normal
MGD3	At2g11810	DAG and UDP-Gal	Plastid outer envelope	Normal
SFR2	At3g06510	Galactoglycerolipid	Plastid outer envelope	Reduced freezing tolerance
DGD1	At3g11670	MGDG and UDP-Gal	Plastid envelope	Reduced DGDG accumulation
DGD2	At4g00550	MGDG and UDP-Gal	Plastid outer envelope	Normal

### DGDG synthases

DGDG is the second most abundant galactolipid and is synthesized by a galactosyltransferase, UDP-galactose:MGDG galactosyltransferase (DGDG synthase) (Dörmann et al., 1995). The first DGDG synthase in Arabidopsis, DGD1, was revealed by isolation of the *dgd1* mutant, which showed 90% reduction in DGDG content (Dörmann et al., 1995). The molecular mapping of the mutation site in *dgd1* revealed that DGD1 encodes a galactosyltransferase with functional DGDG synthase activity (Dörmann et al., 1999). Homology search revealed a second DGDG synthase, DGD2 (Kelly and Dörmann, 2002). Interestingly, DGD2 lacks the N-terminal domain required for insertion of the protein into the envelope membrane, although it encodes functional DGDG synthase. The expression of DGD2 is induced with phosphate starvation, but *dgd2* did not show altered DGDG levels, even under this condition (Kelly and Dörmann, 2002); (Kelly et al., 2003). However, the *dgd1 dgd2* double mutant showed a further reduced *dgd1* level of residual DGDG and no increased level of DGDG with phosphate starvation. Thus, both DGD1 and DGD2 may contribute to the increase in DGDG levels in response to phosphate starvation (Kelly et al., 2003). Of note, trace amounts of DGDG still remained in the *dgd1 dgd2* double mutant, which suggests the existence of a third pathway for DGDG biosynthesis (Kelly et al., 2003).

### GGGT-mediated galactolipid synthesis

A third galactosyltransferase is a processive enzyme resulting in oligogalactolipids such as triGDG or tetraGDG and therefore named galactolipid:galactolipid galactosyltransferase (GGGT). Its activity was initially observed as a major DGDG synthetic activity in isolated chloroplasts or envelope preparations (van Besouw and Wintermans, 1978). However, isolation of DGD1 and DGD2 revealed that UDP-galactose-dependent galactosylation of MGDG is the genuine DGDG synthesis (Kelly et al., 2003). GGGT was revealed in Arabiopdisis with isolation of a series of trigalactosyldiacylglycerol mutants (TGD), *tgd1* to *tgd4* (Xu et al., 2003,2005,2008); (Awai et al., 2006); (Lu et al., 2007). These mutants all accumulate TriGDG under normal growth conditions and are defective in a component of lipid trafficking from the endoplasmic reticulum (ER) to chloroplast. TGD1-3 all localize at the chloroplast envelope, with TGD1 a permease-like protein (Xu et al., 2003,2005), TGD2 a phosphatidic acid-binding protein (Awai et al., 2006); (Lu and Benning, 2009) and TGD3 an ATPase (Lu et al., 2007). Interestingly, TGD4 is localized at the ER and is phosphatidic acid-binding, although *in vivo* interaction with TGD1-3 needs to be shown (Xu et al., 2008); (Wang et al., 2012). Because of their similarity with ABC transporter, these 3 proteins are assumed to assemble a multipartite complex of a lipid transporter mediating lipid trafficking from the ER to chloroplast (Benning, 2008). Accumulation of triGDG is thus considered to be due to the disconnection in lipid trafficking, which could occur when the chloroplast or envelope is isolated.

Because of the odd enzymatic property of GGGT, its identity has long been an issue. Recently, Moellering and the Benning group found that *SENSITIVE TO FREEZING 2*, a gene essential for freezing tolerance in Arabidopsis, encodes a processive galactosyltransferase that produces oligogalactolipids by transferring galactose groups from MGDG (Moellering et al., 2010). Because this reaction yields DAG as a product, the increased DAG level leads to the accumulation of triacylglycerol. This discovery answered the persistent question about galactolipid biosynthesis and opened up a new issue of the link among freezing stress, galactolipid synthesis and triacylglycerol accumulation.

## Galactolipid biosynthesis in floral organs

**Figure 3:**
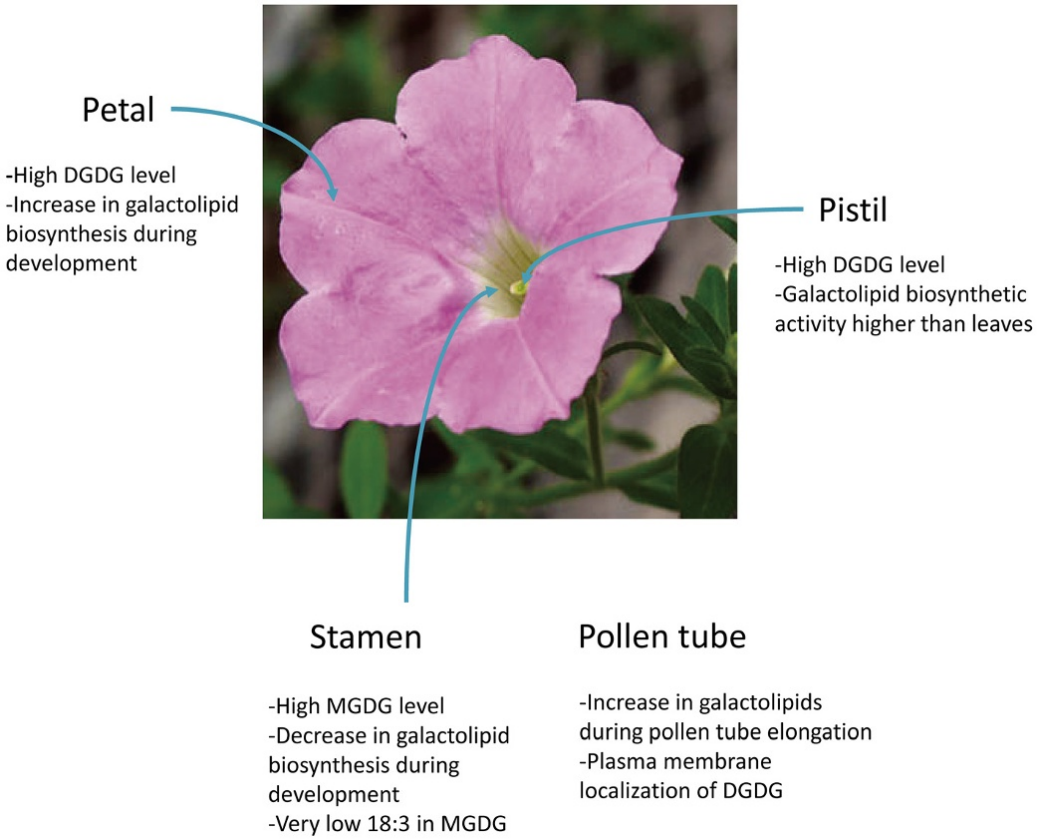
**Schematic illustration of known galactolipid functions in floral organs.**

Given that chloroplasts are differentiated as a form of plastids, an important question was whether galactolipids are unique in chloroplasts or are widely found in different plastids. Biochemical studies found significant levels of MGDG and DGDG in non-photosynthetic organs (Kleinig and Liedvogel, 1978); (Camara et al., 1983); (Alban et al., 1988). Later, Wu and the Huang group discovered and characterized a new type of plastid named elaioplasts from Brassica tapetum, which contains significant levels of galactolipids (Wu et al., 1997; 1999). In agreement with this evidence, type B MGDG synthases are actively expressed in floral organs (Awai et al., 2001); (Kobayashi et al., 2004). This finding prompted us to perform biochemical studies in different floral organs of *Petunia hybrid* to explore yet-unknown roles of galactolipids in flowers (Figure 3) (Nakamura et al., 2003); (Nakamura and Ohta, 2007); (Nakamura et al., 2009).

### Petal

The level of MGDG in petals is about one third of that in leaves (Nakamura et al., 2003). However, the level of DGDG is about one half of that in leaves, for a ratio of MGDG to DGDG of almost 1 (Nakamura et al., 2003). Considering that leaves and stamens contain 1.5- to 2-fold more MGDG than DGDG, petals are characterized as floral organs containing more DGDG (Nakamura et al., 2003). This proportion is well maintained during flower development (Nakamura et al., 2009). As compared with leaves, in petals, MGDG and DGDG both contain less 18:3 but more saturated fatty acids such as 16:0, 18:0 or 18:2 (Nakamura et al., 2003). Galactolipid synthetic activity of total crude extract from petals showed that with a sufficient amount of DAG and UDP-Gal, the activity to produce MGDG is higher in petals than leaves (Nakamura et al., 2003). The activity is still higher in petals without adding DAG, which suggests that petals contain a higher amount of DAG than do leaves (Nakamura et al., 2003). In fact, quantification of DAG levels among floral organs showed that petals, stamens and pistils all contain much higher levels of DAG than do leaves (Nakamura and Ohta, 2007). DGDG synthetic activity is almost the same between petals and leaves but is higher in petals without the addition of exogenous DAG, probably because of higher MGDG synthesis caused by high DAG levels (Nakamura et al., 2003). Of note, during flower development, petals show the greatest increase in both MGDG and DGDG synthesis (nearly 6-fold increase) (Nakamura et al., 2009). This observation suggests that overall galactolipid biosynthesis is highly stimulated during petal development (Nakamura et al., 2003,2009).

### Stamen

Among the 3 floral organs, stamens show the highest MGDG levels and the level is about half that in leaves (Nakamura et al., 2003). However, the ratio of MGDG to DGDG is highly similar in stamens and leaves (Nakamura et al., 2003). The fatty acid composition of DGDG in stamens follows that in petals (i.e., enriched with less-unsaturated fatty acids; (Nakamura et al., 2003). However, the fatty acid composition of MGDG is intriguing: it contains little 18:3 even though MGDG is a major 18:3 source for oxylipin production in plastids (Nakamura et al., 2003). In fact, the Arabidopsis mutant *dad1*, which is deficient in anther dehiscence, is mutated in a gene encoding lipase that liberates 18:3 fatty acid from the glycerlipid backbone for jasmonic acid synthesis (Ishiguro et al., 2001). In Petunia stamen, a major portion of 18:3 in MGDG may be partially converted to a form of oxylipin that is undetectable by the analysis method used to profile fatty acid composition. Indeed, Arabidopsis features an MGDG containing 12-oxophytodienoic acid as an acyl moiety, whose levels are greatly increased with senescence or pathogen attack (Stelmach et al., 2001); (Hisamatsu et al., 2005); (Andersson et al., 2006); (Buseman et al., 2006). How anthesis is achieved in Petunia stamens in the absence of 18:3-containing MGDG is an issue for future investigation. The galactolipid biosynthetic activity in stamens is the lowest among the 3 floral organs (Nakamura et al., 2003). The activity is higher in the earlier stage of flower development and is decreased after full maturation of flowers. (Nakamura et al., 2009).

### Pistil

Pistils are the most unique among the floral organs we discuss in terms of predominant DGDG levels relative to that of MGDG (Nakamura et al., 2003). Indeed, levels of DGDG are almost twice as high as those of MGDG, which suggests that pistil development, together with massive expansion of petal mass, contributes to the overall increase in DGDG level in flowers (Nakamura et al., 2003). The massive upregulation of DGDG synthesis is also supported by an increase in ratio of MGDG to DGDG from 0.25 to 1.13 during flower development (Nakamura et al., 2009). The galactolipid biosynthetic activity in developed pistils is the highest among the floral organs and even higher than that of leaves (Nakamura et al., 2003). Moreover, the activity is much higher without exogenous DAG, which agrees well with high levels of DAG in pistils (Nakamura et al., 2003). That pistils possess galactolipid biosynthetic activity higher than that in photosynthetic tissue such as leaves highlights the significance of galactolipid biosynthesis in non-photosynthetic organs. The fatty acid composition of galactolipids in pistils most resembles that of leaves as compared with petals and stamens (Nakamura et al., 2003).

### Pollen tube

Galactolipids can be utilized even during pollen tube growth. Glycerolipid profiling of lily pollen tubes before and after elongation revealed a 5.7-fold increase in DGDG level and 2.8-fold increase in MGDG level (Nakamura et al., 2009). Use of a specific antibody against DGDG in developing Arabidopsis pollen revealed that the DGDG level is enriched in the peripheral region of the pollen tube, which suggests localization of DGDG at the plasma membrane (Botté et al., 2011). DGDG is not a primary component of the plasma membrane but can replace phospholipids with phosphate starvation (Härtel et al., 2000); (Andersson et al., 2003). Therefore, the developing pollen tube likely uses DGDG instead of phospholipids for rapid plasma-membrane expansion without consuming much of the limited phosphate source.

## Conclusion

Galactolipid biosynthesis is well understood in the model system Arabidopsis. Meanwhile, ample biochemical data from different botanical flowers show intriguing features of galactolipid biosynthesis in floral organs. Although the functional difference between type A and B MGD is clear and type B is more involved in MGDG synthesis in flowers, double knockout of MGD2 and MGD3 does not result in defective flower development nor MGDG level (Kobayashi et al., 2009b). This finding indicates an involvement of MGD1 in flowers, although functional study with an *mgd1* mutant is challenging because it has a lethal phenotype (Kobayashi et al., 2007). Even though Arabidopsis flowers are too tiny for use in lipid biochemistry, advances in genetic study, especially in relation to flower development, facilitates further understanding of the physiological roles of flower lipids. In this regard, a new transgenic system is needed to suppress MGD1 only in flowers with, for example, chemically inducible promoter-driven RNAi to control the spatiotemporal expression profiles of MGDG. Moreover, we need to know the differential distribution of lipid species within the floral organ. An emerging mass spectrometry-based lipid imaging system is allowing for visualizing the distribution of lipid species at the tissue level, although better resolution may be needed for the complex floral tissues of Arabidopsis (Horn et al., 2012). The combination of targeted lipid biosynthetic gene knockout and fine lipid-imaging systems will lead to the next generation of lipid research in flower development.

## Authors’ original submitted files for images

Below are the links to the authors’ original submitted files for images. Authors’ original file for figure 1 Authors’ original file for figure 2 Authors’ original file for figure 3 Authors’ original file for figure 4

## Abbreviations

DAG: *sn*-1,2-diacylglycerol
DGDG: Digalactosyldiacylglycerol
GGGT: Galactolipid:galactolipid galactosyltransferase
MGDG: Monogalactosyldiacylglycerol
TGD: Trigalactosyldiacylglycerol
UDP: Uridine diphosphate.
