# Supplementary figures and images for: Galactolipid biosynthesis in flowers
Source: Bot Stud. 2013 Aug 30;54:29. doi: 10.1186/1999-3110-54-29 (PMC5432751; doi:10.1186/1999-3110-54-29)

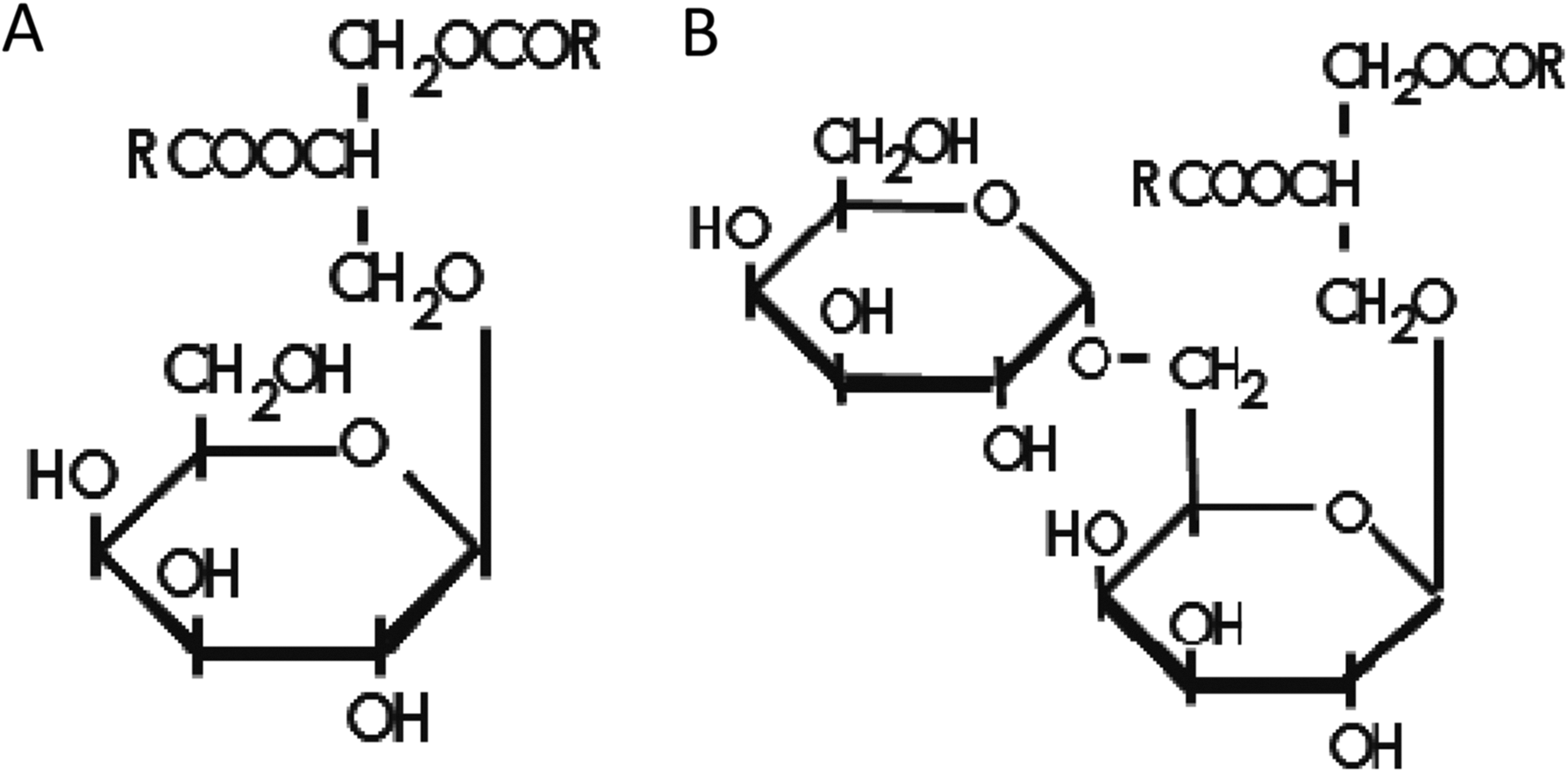

Supplement: Supplementary file 1 — Authors’ original file for figure 1 [file 40529_2012_21_MOESM1_ESM.tif]

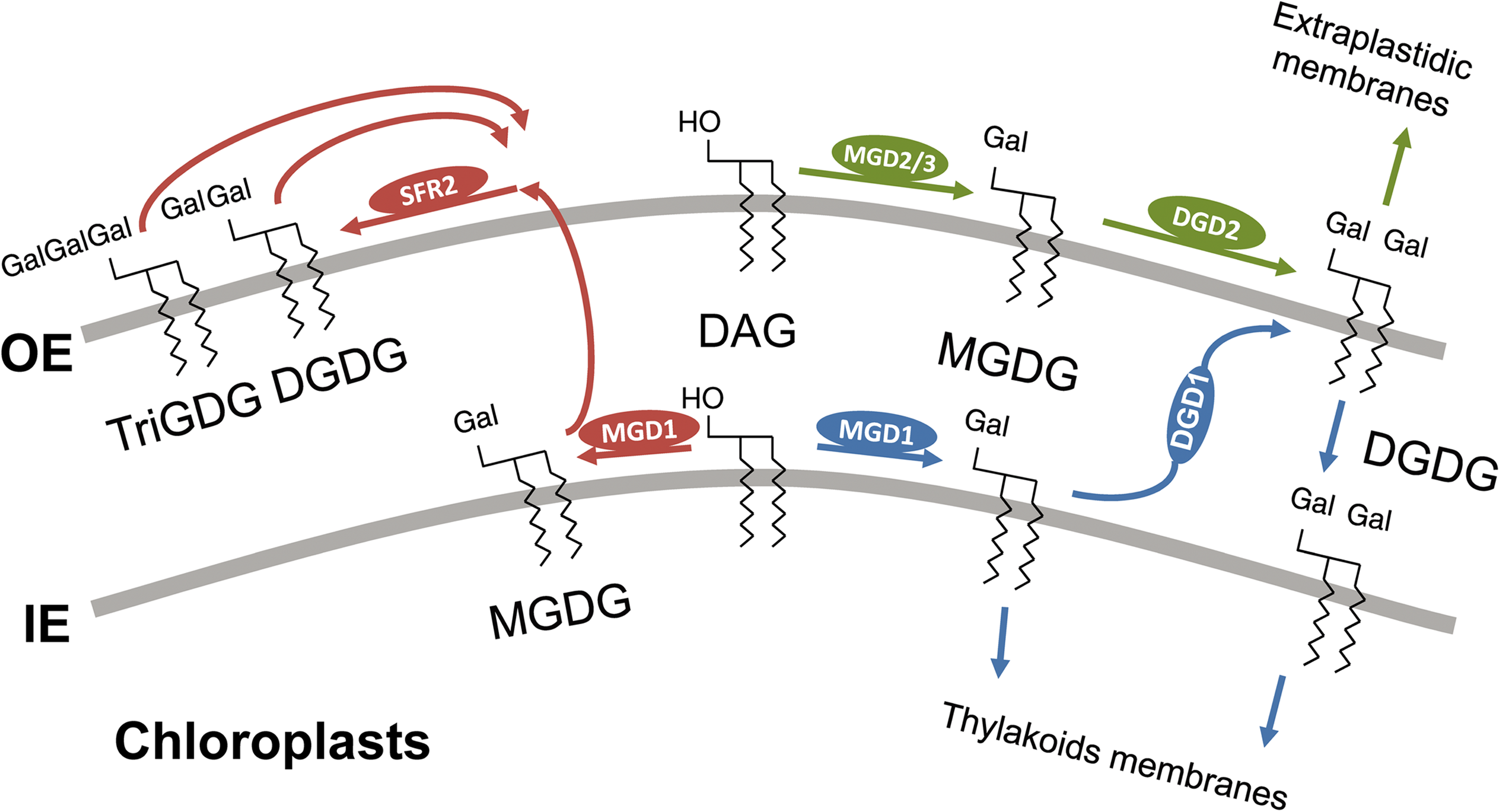

Supplement: Supplementary file 2 — Authors’ original file for figure 2 [file 40529_2012_21_MOESM2_ESM.tif]

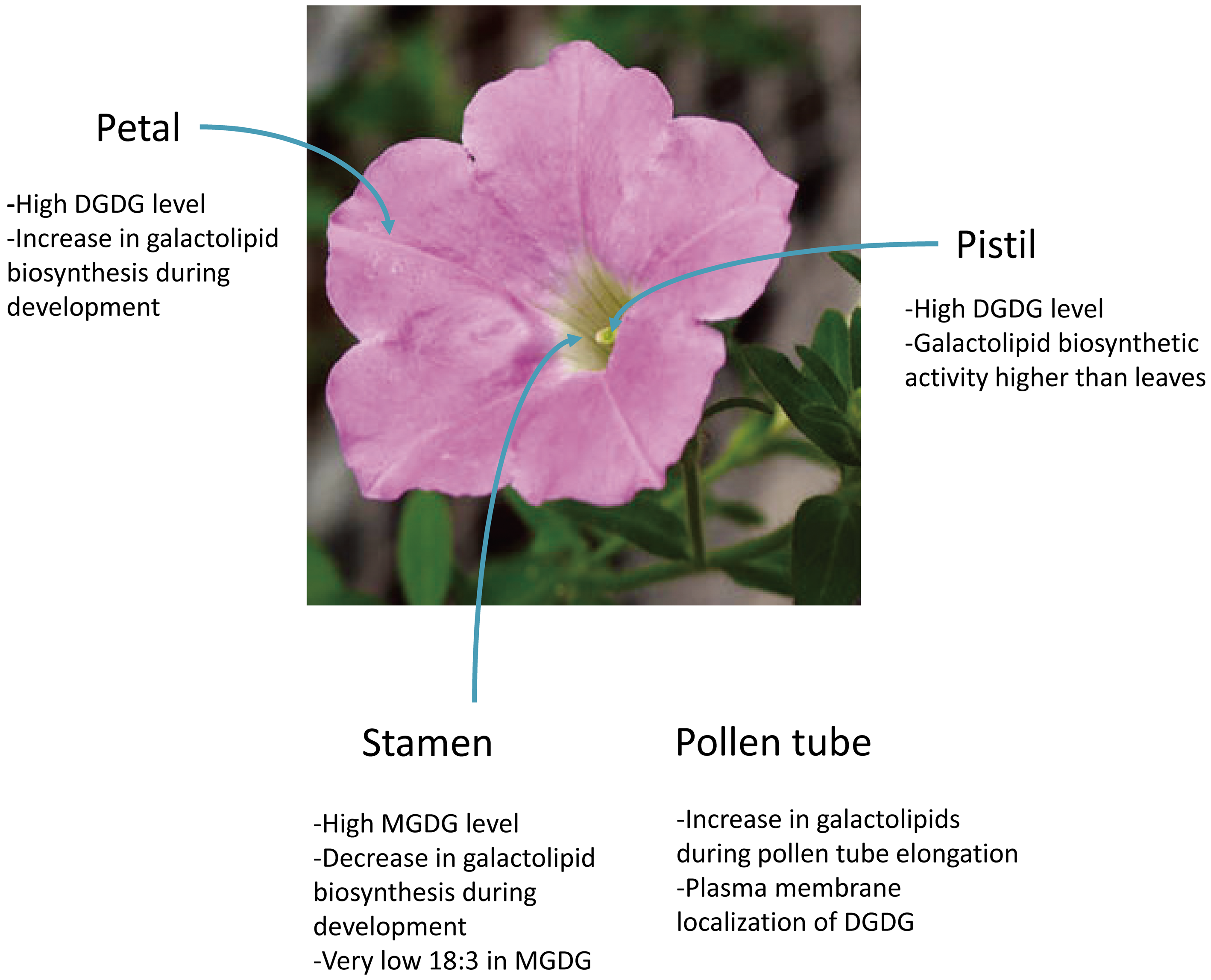

Supplement: Supplementary file 3 — Authors’ original file for figure 3 [file 40529_2012_21_MOESM3_ESM.tif]
